# The influence of childhood parenting style on self in the period of youth: analysis of chain mediation effect on *DanQi*

**DOI:** 10.3389/fpsyg.2025.1621545

**Published:** 2025-08-05

**Authors:** Han Xu, Li Li, Weidong Wang, Xueyu Lv, Jinhua Zhang, Liang Zhang, Kaiyi Huang, Jian Wang

**Affiliations:** ^1^Guang’anmen Hospital, China Academy of Chinese Medical Sciences, Beijing, China; ^2^Department of Medical Nursing, Jiyuan Vocational and Technical College, Jiyuan, China; ^3^Faculty of Chinese Medicine, Macau University of Science and Technology, Macau, China

**Keywords:** parenting style, self, *DanQi*, courage, chain mediation, youth development

## Abstract

**Background:**

Parents’ parenting styles during early development play a crucial role in shaping various psychological attributes. While much research has focused on cognitive development and emotional regulation, the impact on self-concept formation in adolescence has remained underexplored. Additionally, existing studies neglected the mediating role of psychological resources, such as courage, in the intergenerational transmission of traits and risk factors. Courage (defined as mental strength) is integrated into *DanQi,* a TCM concept combining physiological and psychological resilience, providing a cross-cultural perspective.

**Purpose:**

The primary objective of this research is to explore the chain mediation effect of *DanQi* in the relationship between childhood parenting styles and self-concept in young adulthood. We have hypothesized that positive parenting styles during childhood indirectly influence the development of self-concept in young adulthood by fostering elevated levels of *DanQi*—conceptualized as boldness or courage—throughout adolescence and into early adulthood.

**Methods:**

This research employed a quantitative, cross-sectional design, utilizing a stratified random sampling technique to survey urban populations across six major regions of China. Data were collected using subscales from the Wang Weidong Memory-Tracing Personality Developmental Inventory (WMPI), assessing parenting styles, courage, and self-concept across three developmental stages: childhood, adolescence, and young adulthood. Data collection occurred between July and November 2016. A total of 6,539 valid responses were obtained. The statistical software used for data analysis was SPSS 25.0. Data were analyzed using descriptive statistics, correlation, and regression, and mediation testing (PROCESS Model 6).

**Results:**

The findings revealed significant positive associations between childhood parenting styles and *DanQi* in adolescence and young adulthood, as well as self-concept in young adulthood. Mediation analysis indicated that childhood parenting styles indirectly influenced the development of self-concept in young adulthood through adolescent *DanQi* and young adulthood *DanQi*. *DanQi* served as an important mediating factor in the chain of relationships.

**Conclusion:**

This research provides empirical evidence for the mediating role of *DanQi* in linking childhood parenting styles to the development of self-concept, underscoring the importance of early parenting in shaping long-term psychological outcomes.

## 1 Introduction

Childhood experiences — particularly parenting styles **—** are widely recognized as fundamental factors in psychological development. Research has extensively examined how parenting styles affect children’s cognition (Wang et al., 2022), emotion regulation (Ding et al., 2022; Francis et al., 2021), and various psychological traits over time. However, little is known about how these influences shape adolescent self-concept through psychological resources (e.g., courage). Most previous studies focused on isolated developmental periods, especially adolescence, neglecting the enduring impact of childhood parenting styles. This gap limits our comprehensive understanding of how parenting approaches affect development across different life stages. Adolescence is a critical time for forming self-concept, including a sense of self-awareness, social roles, and future aspirations. Research on the effects of early parenting on adolescent self-identity is crucial because many aspects of psychological growth and development may be influenced by early parenting and may have lasting impacts on psychological development.

Empirical research has consistently demonstrated that parenting styles in childhood impact psychological development (Jiang et al., 2024; Kılıçkaya et al., 2023; Yan et al., 2024). Although existing research shows how parenting styles have direct effects on children’s psychological traits, it neglects psychological strengths, such as courage, that may mediate cycles across generations. Furthermore, concepts specific to cultures are hardly included in such analyses. Western psychology has narrowly defined courage either as the ability to overcome and confront fear (Jena et al., 2021; Konter et al., 2020) or to regulate emotions in the face of threat (Pajestka and Skałacka, 2025; Perkins et al., 2022). Such a limited view tends to overlook the complex mind–body interactions highlighted in cross-cultural perspectives. Traditional Chinese medicine (TCM) offers the concept of *DanQi*, a complex resource incorporating physiological function and psychological resilience. This construct complements and extends existing theory, providing a more comprehensive explanation of the long-term effects of early parenting than monocultural frameworks.

### 1.1 Theoretical framework of parenting styles and self-development

The TCM Personality Development System (Zhang et al., 2022), developed by Dr. Weidong Wang, combines holistic philosophy with Confucian-Taoist cultural values to provide a framework for understanding psychosocial development.

Wang’s model, which aligned with Vygotsky’s sociocultural theory (Vasileva and Balyasnikova, 2019), organizes development into three key stages: early development (birth to age 7), identity formation (8–18 years), and social integration (19–25 years) (Wang et al., 2016a). According to this theory, internal factors of personality development were categorized into seven components: *DanQi*, self, cognitive style, willpower, interpersonal relationships, sexual development, and worldview. The two primary external environmental factors influencing children’s psychological development were considered to be parenting styles and adverse life experiences. Wang’s understanding of parenting styles closely aligned with Baumrind’s two-dimensional model, which differentiates between emotional support and control in parenting (Kılıçkaya et al., 2023). For example, Authoritative parenting combines high responsiveness with firm expectations, fostering emotional security and self-control. Conversely, authoritarian parenting emphasizes control with little emotional support, leading to low self-esteem and dependency in children. Neglectful parenting, characterized by low emotional involvement, can result in emotional alienation and social problems.

Early parenting exerts lasting effects on an individual’s psychosocial development, which can influence self-concept and mental resilience throughout life, ultimately forming the core of personality development. Bowlby’s attachment theory (Holmes, 2013; Sroufe, 1986) emphasizes the importance of early parent–child interactions in fostering emotional trust and a coherent sense of self. A secure emotional foundation, established through stable caregiver relationships, enables children to integrate new experiences and maintain a coherent sense of self as they mature. Erikson’s psychosocial development theory (Erikson, 1993) further posits that adolescence is a critical period for identity formation, with family support serving as a secure base for adolescents to explore and integrate their self-concept. Self-conceptualization involves updating cognitive schemas, where individuals integrate social expectations with internal needs, transforming self-perception from fragmentation to systematization (Garcia et al., 2023). This process deepens awareness of roles and connects social norms with personal goals. Parental support, by promoting moderate autonomy, stimulates cognitive development and helps sustain self-efficacy, preventing both overprotection and permissiveness. Therefore, a balanced approach to parenting is crucial for developing a well-adjusted sense of self.

### 1.2 Security, courage, and *DanQi*

A sense of security forms the cornerstone for courage and boldness, providing an essential psychological resource for individuals. From a developmental perspective, the formation of a sense of safety is grounded in a dual theoretical framework. In the vertical dimension of the hierarchy of needs, Maslow’s theory demonstrates the central position of safety as a fundamental need. When physiological needs are satisfied and existential threats no longer constrain individuals, establishing a sense of security (the second level of needs) becomes a prerequisite for pursuing higher-order psychological development (Maslow, 1943). The horizontal dimension can be explained through attachment theory. Bowlby posits that basic trust in the world is developed by children through stable attachment relationships. This security foundation enables individuals to redirect cognitive resources from threat monitoring toward environmental exploration, predicated on perceptions of safety, authenticity, and freedom (Badouk-Epstein and Yellin, 2018). Only when psychological security needs are fulfilled can individuals effectively cope with stress and challenges. Subsequently, these individuals can pursue higher-level needs (such as belonging, respect, and self-actualization) and possess sufficient psychological resources to confront difficulties. This sense of security facilitates emotional regulation and stress management, thereby establishing the foundation for courage development. In the absence of security, individuals frequently experience elevated levels of anxiety and fear, which inhibits their capacity to demonstrate sufficient courage when confronted with crises during adversity.

Courage can be conceptualized as an individual response to uncertainty and risk that provides security, stability, and self-confidence (Abdollahi et al., 2022). Courage functions as a protective mechanism for coping with fears that are encountered during daily challenges and within contemporary social contexts (Santilli et al., 2021). Courage represents the capacity to remain resolute when confronted with fear while maintaining unwavering commitment to valued principles (Ajadi, 2024). This construct encompasses physical, moral, and psychological dimensions of courage (Abdollahi et al., 2022; Santilli et al., 2021). Courage typically involves a deliberate process that is contingent upon both the individual and the specific task or situation with which they are confronted. This process necessitates the selection of what is deemed important, which actions should be undertaken, and how to proceed with life in a personally meaningful manner (Chowkase et al., 2024). Courage serves as a “trigger for action,” compelling individuals to translate value judgments into tangible behavioral responses. In this model, courage is conceptualized by the balancing of potential risk costs against moral commitments (Ruixin et al., 2024). Thus, courage is predicated on a sense of safety. Because of this sense of safety, we can avoid or neutralize fear and initiate action, surmounting the fear that would otherwise paralyze us and enabling us to progress forward. To the degree that we are not safe and do not expect uncertainty to yield benefits, we will avoid uncertainty or not be courageous.

The concept of *DanQi* from TCM is broader than that of courage from a cultural perspective. Although the cultural background and theoretical foundation are vastly different (Western psychology and TCM, respectively), their functions are comparable in describing courage under uncertainty. The concept of *DanQi* in TCM carries numerous metaphors. These metaphors represent both external mobility and internal stability. Such usage of metaphors is consistent with the results from Conceptual Metaphor Theory (Zwaan, 2021). In TCM, *DanQi* is a comprehensive concept including both physiological and psychological concepts. In modern psychology, it includes traits such as risk-taking, the tendency to make quick decisions when faced with uncertainty, and the tendency to persevere in one’s moral convictions and mental toughness. In general, individuals with sufficient *DanQi* are expected to be courageous and bold, remaining calm under pressure and taking action when facing challenges.

From a physiological viewpoint, *DanQi* is associated with the function of the gallbladder. The gallbladder operates in conjunction with the liver to aid in digestion and overall health. Activation of the body’s energy is regulated by the function of *DanQi* in the body. From a psychological viewpoint, the ability to make judgments and decisions under situations of uncertainty is crucial. It involves making quick decisions in situations of risk and taking responsibility for the outcome when complete information is not available.

In TCM, an individual with abundant *DanQi* remains calm and decisive under pressure, mirroring the concept of “mental toughness” in modern psychology. Additionally, *DanQi* represents social resilience in Chinese culture. For example, the Chinese idiom “the liver and gallbladder reflect each other” translates to “being open and sincere with one another” in English. This idiom conveys loyalty and trust, suggesting that social support is essential for strengthening courage, which, in turn, helps individuals adapt to their social environment and promote self-development. This mind–body unity theory aligns with the concept of “embodied cognition” in modern psychology (Shapiro and Stolz, 2019; Zwaan, 2021). This cognitive process relies on sensory, motor, and emotional systems and involves the expression of mental imagery (Marre et al., 2021). This paper focuses on the psychological aspect of the concept of *DanQi*.

### 1.3 Research hypothesis

While extensive research has explored the effects of parenting approaches on children’s developmental outcomes, fewer studies have focused on how these early influences shape later stages, particularly in fostering *DanQi* during adolescence. Theories on self-concept and identity development, such as Erikson’s Stages of Psychosocial Development (Erikson, 1993), highlight adolescence as a critical period for shaping identity. However, these theories have not adequately addressed the role of grit in fostering resilience and a positive self-concept during adolescence.

This study aims to fill this gap by investigating how *DanQi* acts as a mediator within the connection between childhood parenting approaches and adult self-concept. Drawing on Prof. Weidong Wang’s theory of personality system development, the study hypothesizes that childhood parenting styles indirectly affect adult self-concept through their influence on *DanQi* during adolescence and young adulthood.

Specifically, it is hypothesized that positive parenting styles promote higher levels of *DanQi* during adolescence and young adulthood, thereby enhancing self-concept in young adulthood.

*H1:* Childhood parenting styles directly influence the development of the young adult self.

*H2:* Childhood parenting styles influence the young adult self indirectly through their effect on *DanQi* levels during adolescence

*H3:* Childhood parenting styles influence the young adult self indirectly through their effect on *DanQi* levels during young adulthood.

*H4:* Childhood parenting styles influence *DanQi* in adolescence. Adolescent *DanQi* subsequently affects *DanQi* in young-adulthood. Young adult *DanQi* ultimately influences the young adult self.

**Figure 1:**
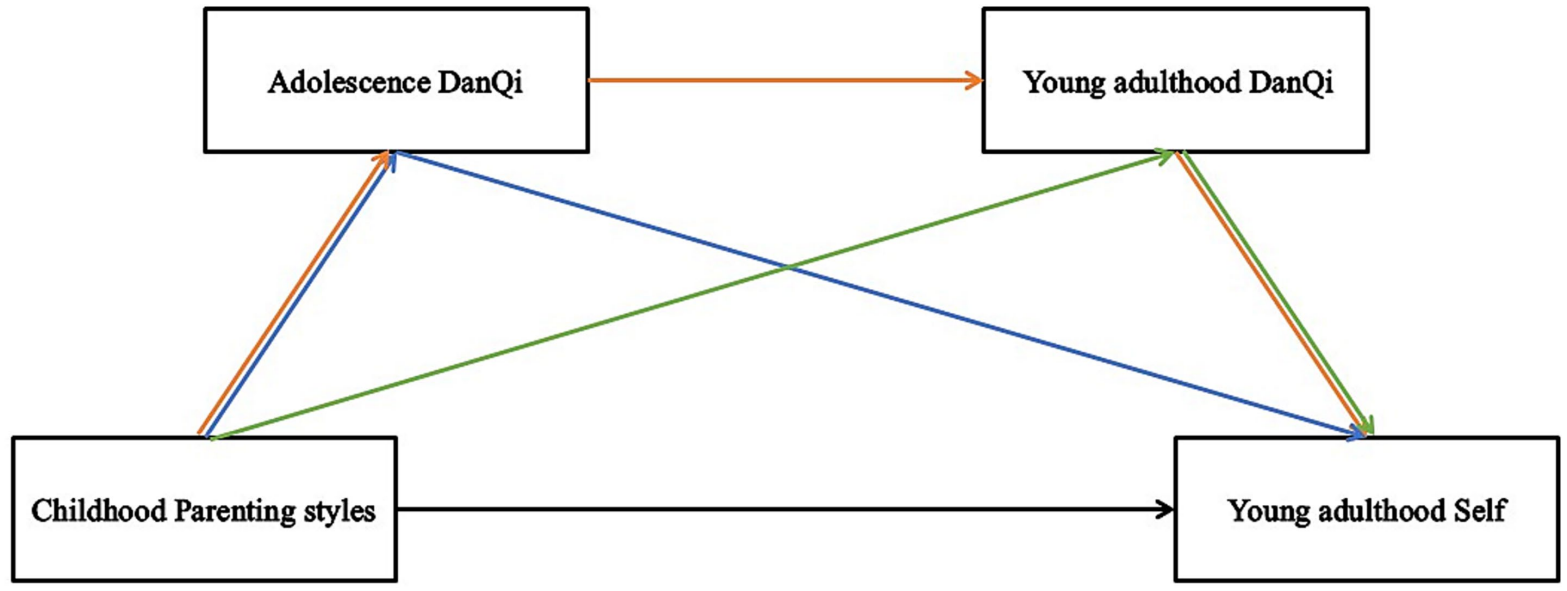
Assumption model. Hypothesis 1 is the black line. Hypothesis 2 is the blue line. Hypothesis 3 is the green line. Hypothesis 4 is the orange line.

These hypotheses are illustrated in Figure 1, where the relationships between childhood parenting styles, *DanQi* at different stages, and young adulthood self-concept are depicted. By examining *DanQi*’s mediating effects, this study provides new insights into how early life experiences shape later psychological outcomes. Additionally, this study integrates Eastern and Western psychological theories alongside TCM theories. It emphasizes the importance of cultivating *DanQi* during childhood and adolescence to foster a positive self-concept in young adulthood. This cross-cultural perspective offers valuable insights into how parenting strategies shape psychological resources, such as *DanQi* and courage, ultimately influencing an individual’s self-concept and ability to navigate the challenges of adult life.

## 2 Research methodology

### 2.1 Research design

This quantitative, cross-sectional study examines the relationship between childhood parenting styles, *DanQi*, and self-concept development during adolescence, based on personal recollections. Data were gathered through self-reported questionnaires, with a stratified random sampling method employed to ensure representation across different urban regions of China. The study aims to investigate how early parenting styles influence the current state of psychological resources, such as courage and self-concept, and explores the mediating role of *DanQi* across different developmental stages.

### 2.2 Research tools

**Figure 2:**
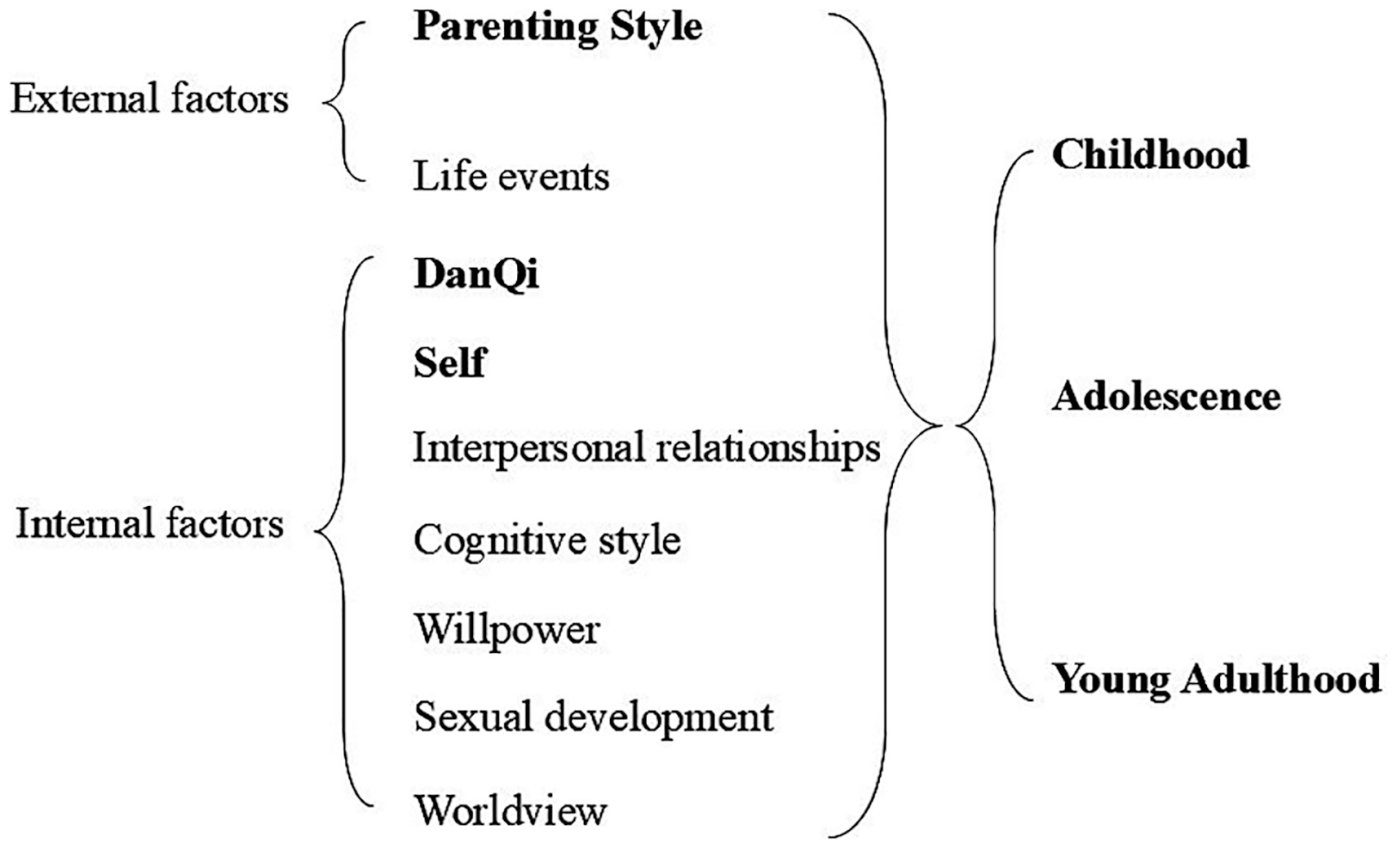
WMPI scale structure.

The Wang Weidong Memory-Tracing Personality Development Inventory (WMPI), developed by Prof. Wang Weidong’s team (Wang et al., 2016b; Xu et al., 2022), was employed. This inventory is used to assess an individual’s personality traits by asking them to recall their personal growth experiences and understand how these experiences shape their perception. The inventory employs a five-point Likert scale, where 1 represents “never,” 2 represents “sometimes,” 3 represents “often,” 4 represents “most of the time,” and 5 represents “always.” The questionnaire consists of nine subscales, categorized into internal and external elements. The external elements include the subscales of parenting style and life events. In contrast, the internal elements consist of seven subscales: *DanQi*, ego, willpower, interpersonal interactions, thinking styles, worldview, and sexual development. Each subscale includes multiple questions, except for the worldview and sexual development subscales, which cover only adolescence and adulthood. The remaining subscales address childhood (ages 3–6), adolescence (ages 7–18), and young adulthood (ages 19–25). The specific structure of the scale is shown in Figure 2. In this study, the subscales for Childhood Parenting Styles, Adolescent *DanQi*, Young Adulthood *DanQi*, and Young Adulthood Self were included.

The Parenting Styles subscale is divided into six dimensions: punishment, severity, over-involvement, indulgence, inconsistent education, and neglectful protection. The following provides a detailed explanation of how these aspects are assessed: The punishment evaluates the extent to which parents use harsh disciplinary methods. This dimension includes items such as “My parents punish me severely” and “My parents punish me without cause.” The severity evaluates the severity of parental behavior. Items such as “I am afraid of my mother because she is very strict” and “I feel intimidated by my father’s harshness” reflect this dimension. The over-involvement assesses the extent to which parents exert control over personal decisions. This dimension includes items such as “My parents must handle everything I do” and “My parents choose my friends for me.” The indulgence reflects excessive pampering, including items such as “When I am bullied or criticized, my parents protect me” and “My parents fulfill all my wishes.” The inconsistent education highlights inconsistencies in parental behavior, such as “My father is lenient, but my mother is strict,” and “My parents have inconsistent expectations for the same behavior.” The neglectful protection evaluates neglectful behavior, including items such as “When I need support, my parents ignore me” and “My parents forget to fulfill their promises to me.” This section includes 30 items, with each item corresponding to three distinct developmental phases. Participants provide responses based on their perceptions of parenting styles at each stage. The internal consistency alpha coefficient for the Childhood Parenting Styles subscale is 0.899.

The *DanQi* subscale comprises four dimensions: interpersonal fear, nature fear, adaptability, and inexplicable fear. Each dimension is assessed based on participants’ emotional responses to relevant situations. The following provides a detailed description of how these aspects are measured: The interpersonal fear measures anxiety and fear in social situations. Items such as “I am afraid to do unfamiliar things in front of others” and “I avoid arguing with others” are included. The natural fear primarily measures an individual’s fear response to natural phenomena. Items such as “I am afraid of thunderstorms” and “I am afraid of the dark” are included. The adaptability evaluates an individual’s coping mechanisms under stress and in the face of uncertainty. Items such as “I cannot tolerate unfairness” and “I want others to do as I think they should” are included. The inexplicable fear measures fear and anxiety when facing uncertainty. Items such as “I feel uneasy and unsettled” and “I worry that something unfortunate will happen” are included. The subscale contains 29 items, each corresponding to three distinct periods. Participants recall their states at various time points to answer the items. The internal consistency alpha coefficients for the Adolescent and Youth Boldness subscales were 0.871 and 0.870, respectively.

The Self subscale includes five dimensions: social self, physical self, family self, self-determination, and self-care. The following explains how these aspects are evaluated: The social self measures individuals’ feelings of acceptance and trustworthiness in social situations. Items such as “I feel that others do not want to work with me” and “I find it hard to earn others’ trust” are included. The physical self evaluates feelings about one’s physical appearance and health, such as “I feel my body is not as good as others” and “I feel physically weak.” The family self examines individuals’ perceptions of their place in the family, with items such as “I feel that I am not important in my family” and “My parents love me.” The self-determination focuses on individuals’ independence and decision-making ability, such as “I care too much about what others think of me” and “I must listen to my parents about marriage matters.” The self-care assesses individuals’ ability to take responsibility for their actions and decisions, including items such as “I do not help adults with chores” and “I think I do not have to do anything at home except study or play.” The subscale consists of 21 items, each corresponding to three distinct periods. Participants recall their status at various time points to answer the items. The internal consistency alpha coefficient for the Self subscale in adolescents is 0.832.

### 2.3 Participants

The participants’ data for this study were obtained from the WMPI normative sample databases. Data collection for the Chinese urban population began in late July 2016. The testing of the complete Chinese urban population normative data set was completed over more than four months. The research employed stratified random sampling to survey the urban population across seven distinct regions of mainland China: the Northeast, North, East, Central, South, Southwest, and Northwest. The age groups were 25–34, 35–44, 45–54, 55–64, and 65–75 years old, and the literacy levels were junior high school and below, senior high school, college, university, and graduate school.

**Inclusion criteria** for participants were permanent residents of the study area who:

1. Were between 20 and 70 years;
2. Were not professionals in psychology or mental health;
3. Had not participated in personality-related psychological tests within six months before the interview;
4. Were capable of reading and understanding the content of the questionnaire;
5. Had at least a junior high school education;
6. Had normal cognitive function;

**Exclusion criteria** for participants were:

1. Schizophrenia or other psychotic disorders;
2. Diagnosis of intellectual disability or pervasive developmental disorders;
3. Had been taking antipsychotic, antidepressant, anxiolytic, or cognitive-enhancing medications;
4. Had taken anesthetic medications for pain relief in the past 72 h;
5. A history of alcohol or substance abuse lasting over ten years;
6. Use of any psychoactive substances within three days before the test;
7. Consumption of more than four standard alcoholic drinks per day within three days before the test;
8. Inability to understand Chinese, thus hindering full comprehension of the test instructions.

**Cleaning data criteria** were as follows:

1. Lie detection score above 18;
2. Response time shorter than 40 min or longer than 90 min;
3. A score difference of 3 or more on the retest of the Youth Version of the Questionnaire.

Psychometric studies have demonstrated that a sample size of 2,000 to 3,000 participants is typically required for national norms (Lv et al., 2023; Tan et al., 2025). Therefore, this study required at least 3,000 valid samples. 9,124 samples were ultimately collected, with 6,539 valid samples obtained after data cleaning, resulting in an effective rate of 71.67%.

### 2.4 Data processing

A professional Chinese statistical agency administered the online survey questionnaires. The agency was responsible for participant recruitment and data collection. It strictly adhered to the specified regional sampling requirements, as well as the inclusion and exclusion criteria, to establish a standardized normative database.

Subsequently, after applying rigorous data cleaning criteria, the statistical software SPSS 25.0 was used to perform various analyses, including descriptive statistics, correlation analysis, and regression analysis, aimed at examining the data. Model 6, developed by Andrew F. Hayes (Hayes, 2018), within the PROCESS program, was used to assess the mediation model. The significance of the mediation effect was further evaluated using the bias-corrected nonparametric percentile bootstrap method.

## 3 Results

### 3.1 Descriptive statistics for general information

An overall total of 6,539 reliable responses were obtained in this study. The gender distribution was nearly equal, with males comprising 49.46% and females 50.54%. The age distribution was broad, with the largest proportion (31.73%) in the 30–40 age group, followed by 22.54% in the 40–50 age group, 21.70% in the 20–30 age group, 14.86% in the 50–60 age group, and 9.16% in the 60–75 age group. Regarding ethnic composition, Han Chinese comprised the majority at 96.82%, while ethnic minorities accounted for 3.18%, reflecting the ethnic distribution characteristics of China. In terms of education, 52.18% of the participants had completed junior high school or lower, 22.79% had completed high school, 14.60% had received vocational education, and 10.41% held a bachelor’s degree or higher. As for marital status, 84.78% were married, and 15.22% were in other marital statuses. Regarding siblings, 41.98% of the participants had three or more siblings, 31.12% had two, and 26.79% had only one. Regarding family ranking, 27.65% were the eldest child, 24.94% were the youngest, 20.61% were the middle child, and 26.79% were the only child. Detailed results are presented in Table 1.

**Table 1:** General material analysis.

Characteristic		N	%
Gender	Male	3,234	49.46
	Female	3,305	50.54
Age	(20, 30)	1,419	21.70
	(30, 40)	2075	31.73
	(40, 50)	1,474	22.54
	(50, 60)	972	14.86
	(60, 75)	599	9.16
Nation	The Han nationality	6,331	96.82
	Minority nationality	208	3.18
Educational level	Junior high school and below	3,412	52.18
	High school	1,490	22.79
	Specialist	955	14.60
	Undergraduate or above	681	10.41
Marital situation	Married	5,544	84.78
	Other	995	15.22
Number of brothers and sisters	One	1752	26.79
	Two	2035	31.12
	Three or more	2,745	41.98
Ranking at home	Only child	1752	26.79
	Ranking leader	1808	27.65
	Middle of ranking	1,348	20.61
	Ranking minimum	1,631	24.94

### 3.2 Correlation analysis

A strong positive relationship was identified in all pairwise comparisons between the childhood parenting styles subscale, the adolescent *DanQi* subscale, the young adulthood *DanQi* subscale, and the young adulthood self-subscale. The correlation coefficient between childhood parenting styles and adolescent *DanQi* was 0.202 (*p* < 0.001), signifying a meaningful positive association. The correlation between adolescent *DanQi* and young adulthood *DanQi* was 0.882 (*p* < 0.001), indicating a strong positive connection between the two. The correlation coefficient between young adulthood *DanQi* and young adulthood self was 0.811 (*p* < 0.001), indicating a substantial positive relationship between the two. Moreover, childhood parenting styles showed a significant positive association with young adulthood *DanQi* (0.187, *p* < 0.001) and young adulthood self (0.169, *p* < 0.001). These findings suggest that parenting styles may influence self-identity development by fostering *DanQi*. The detailed correlation coefficients and their levels of significance are presented in Table 2.

**Table 2:** Correlation analysis.

Variable	M [Q1, Q3]	1. Childhood parenting styles	2. Adolescence *DanQi*	3. Young adulthood *DanQi*	4. Young adulthood Self
1. Childhood parenting styles	37 [23,49]	1			
2. Adolescence *DanQi*	55 [44,65]	0.200^***^	1		
3. Young adulthood *DanQi*	52 [42,62]	0.186^***^	0.882^***^	1	
4. Young adulthood Self	34 [27,42]	0.166^***^	0.750^***^	0.811^***^	1

^*^p < 0. 05; ^**^p < 0. 01; ^***^p < 0. 001.

### 3.3 Mediation effect test

**Figure 3:**
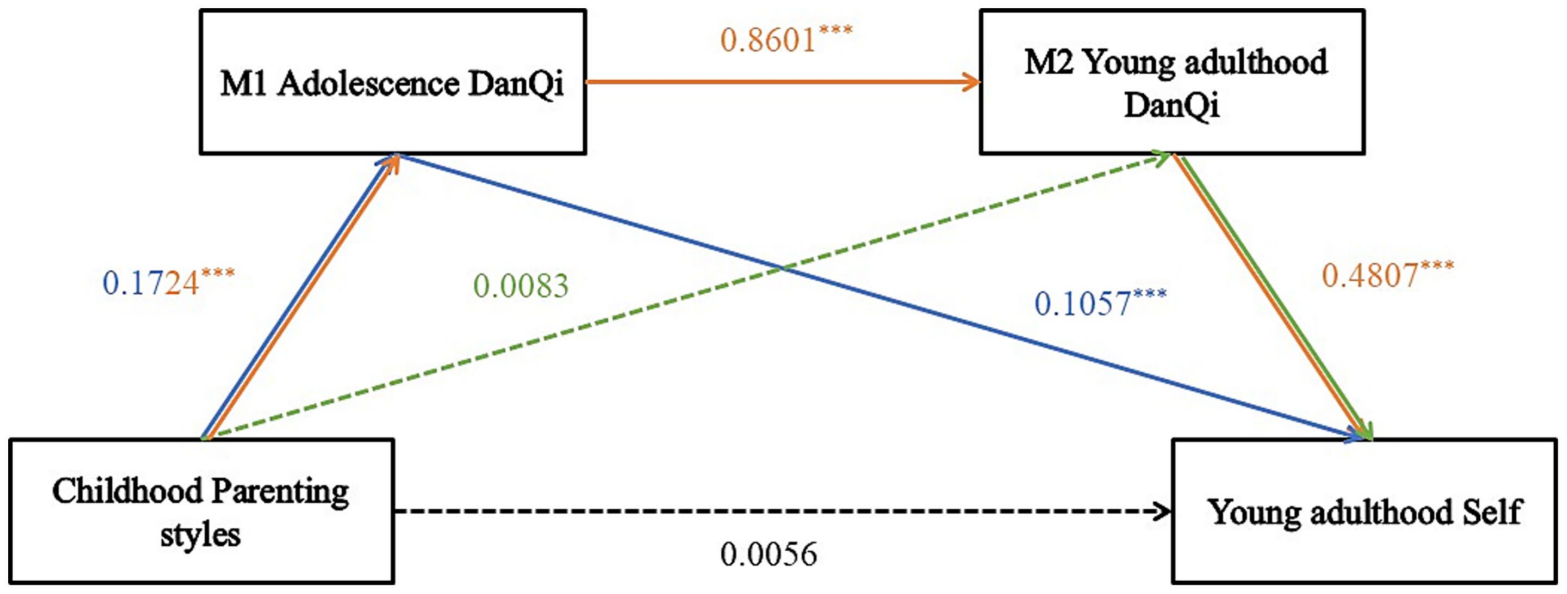
The effects of childhood parenting styles on the Young adulthood self: the chain mediation effect of *DanQi.* Path1 (Hypothesis 2) is the blue line. Path2 (Hypothesis 3) is the green line. Path3 (Hypothesis 4) is the orange line. Solid lines: Represent statistically significant path relationships (*p* < 0.05). Dashed lines: Represent statistically insignificant path relationships (*p* ≥ 0.05).

As presented in Table 3, mediation effects were analyzed using the SPSS macro program PROCESS, developed by Hayes, to examine the mediating role of adolescent and young adulthood *DanQi* between childhood parenting styles and young adulthood self, controlling for gender and age. Regression analyses showed that childhood parenting style directly influenced adolescent *DanQi* (*β* = 0.1724, *p* < 0.001). Adolescent *DanQi* was a significant positive predictor of young adulthood *DanQi* (*β* = 0.8601, *p* < 0.001). Childhood parenting style did not significantly predict *DanQi* in young adulthood (*β* = 0.0083, *p* > 0.05). When predicting young adulthood self, the variables of childhood parenting style, adolescent *DanQi*, and young adulthood *DanQi* were all simultaneously considered. The results showed that both adolescent *DanQi* (*β* = 0.1057, *p* < 0.001) and young adulthood *DanQi* (*β* = 0.4807, *p* < 0.001) acted as significant positive predictors of self-concept in young adulthood. Childhood parenting style was not a significant direct predictor of young adulthood self (*β* = 0.0056, *p* > 0.05). The level of statistical significance of the mediation effect, as shown in Table 4, was assessed using the bias-corrected nonparametric percentile bootstrap technique. The results indicated that childhood parenting style was not a significant direct predictor of young adulthood self (*p* > 0.05). However, the mediating effects of adolescent *DanQi* and young adulthood *DanQi* were significant. As presented in the table, the mediating effects resulted from two mediation chains. First, the indirect effect (Path 1) generated by the pathway Childhood parenting style → Adolescent *DanQi* → Young adulthood Self, with an effect value of 0.0302, accounting for 19.48%. Second, the indirect effect (Path 3) generated by the pathway Childhood Parenting Style →Adolescence *DanQi* → Young adulthood *DanQi* → Young adulthood Self, with an effect value of 0.1182, accounting for 76.26%. The 95% confidence interval derived from the bootstrap method did not encompass 0, indicating that adolescent *DanQi* and young adulthood *DanQi* serve as significant mediators in the pathway linking childhood parenting style to young adulthood self. In contrast, the pathway Childhood parenting style → Young adulthood *DanQi* → Young adulthood self produced an indirect effect (Path 2) of 0.0066, with the bootstrap 95% confidence interval including 0, suggesting that this mediating effect was not significant. The specific pathway through which childhood parenting styles affect young adulthood self is presented in Figure 3.

**Table 3:** Regression analysis between variables.

Regression equation		Overall fit index			Significance of regression coefficient			
Result variable	Predictive variable	*R*	*R*^2^	*F*	*β*	*SE*	*t*	*p*
M1: Adolescence *DanQi*		0.2153	0.0463	105.8377^***^				< 0.001
	Gender				2.2618	0.3853	5.8701^***^	< 0.001
	Age				0.0432	0.0159	2.7079^**^	0.0068
	Childhood parenting styles				0.1724	0.0106	16.3237^***^	< 0.001
M2: Young adulthood *DanQi*		0.8824	0.7787	5746.5443^***^				< 0.001
	Gender				0.8862	0.1824	4.8587^***^	< 0.001
	Age				0.0012	0.0075	0.1633	0.8703
	Childhood parenting styles				0.0083	0.0051	1.6282	0.1035
	M1: Adolescence *DanQi*				0.8601	0.0058	147.2754^***^	< 0.001
Young adulthood self		0.8205	0.6732	2691.2305^***^				< 0.001
	Gender				−2.0956	0.1565	−13.3901^***^	< 0.001
	Age				0.0306	0.0065	4.7507^***^	< 0.001
	Childhood parenting styles				0.0056	0.0044	1.2822	0.1998
	M1: Adolescence *DanQi*				0.1057	0.0104	10.1635^***^	< 0.001
	M2: Young adulthood Self				0.4807	0.0106	45.3678^***^	< 0.001

^*^p < 0.05; ^**^p < 0.01; ^***^p < 0.001.

**Table 4:** The mediating effect of adolescence and young adulthood *DanQi* on childhood parenting styles and young adulthood self.

Path	Effect value	Bootstrap	Bootstrap 95% interval		Relative mediation Effect ratio
			Bootllci	bootulci	
Total indirect effect	0.155	0.0103	0.1352	0.1749	
1. Childhood parenting styles → Adolescence *DanQi* → Young adulthood self	0.0302	0.0036	0.0235	0.0377	19.48%
2. Childhood parenting styles →Young adulthood *DanQi* → Young adulthood self	0.0066	0.0047	−0.0024	0.0159	4.26%
3. Childhood parenting styles →Adolescence *DanQi* → Young adulthood *DanQi* → Young adulthood self	0.1182	0.0079	0.1026	0.134	76.26%

## 4 Discussions

This research investigates the influence of childhood parental influence on personal development during young adulthood, with a particular emphasis on the mediating function of *DanQi* within this process. *DanQi* is not only an important psychological resource in the face of uncertainty and challenge, but also a central element in the formation of self-identity. The current investigation revealed no statistically significant direct association between childhood parenting styles and self-concept in young adulthood. However, the mediation analysis indicates that parenting styles during childhood indirectly influence young adults’ self-concept by affecting their *DanQi* during adolescence and early adulthood. This process involves two pathways: Pathway 1: Parenting styles during childhood influence adolescents’ *DanQi*, which subsequently influences young adults’ self-concept. Pathway 2: Childhood parenting styles influence adolescents’ *DanQi*, and this effect continues to influence young adults’ *DanQi*, ultimately shaping the self in early adulthood. This suggests that parenting styles alone did not have a direct impact on self-perception but instead influenced it indirectly through the mediating effect of *DanQi*, aligning with prior research (Chang et al., 2023).

This mediating role mirrors the “secure base “mechanism(Waters and Roisman, 2019) in Attachment Theory. Self-concept is not directly instilled; rather, it is facilitated by the accumulation of psychological resources through emotional support and autonomy provided by parents (the object of secure attachment) during early development. This helps children build psychological stability, coping skills, and enhances their sense of security and decisiveness. Parents serve as a safe haven in children’s development, providing comfort and support when children feel threatened or distressed(George and Wargo Aikins, 2023). If children are exposed to anger or anxiety expressed by their parents during childhood, it may affect their sensitivity to fear and their ability to regulate it (Woodward et al., 2020).

In this context, the individual’s *DanQi* can be activated. This process fosters confidence in one’s abilities as the individual matures. This confidence not only builds self-esteem but also strengthens self-efficacy, enabling individuals to make decisions and take action more autonomously. When individuals feel threatened or anxious, activating the safety mechanism significantly enhances positive emotions, reduces negative emotions. It provides emotional comfort and helps individuals return to a stable emotional state(Rowe et al., 2020). Within the framework of TCM, activating the safety mechanism reflects “abundant *DanQi*.” Physiologically, this can be interpreted as the restoration of balance in the autonomic nervous system. Psychologically, it is interpreted as a decrease in the tendency to avoid risky decisions, replaced by goal-oriented, decisive, and courageous behavior.

This study also reveals that *DanQi*, as a fundamental psychological resource, remains present throughout the lifespan and intensifies over time. Closely associated with self-perception, emotion regulation, and behavioral responses, *DanQi* serves not only as a source of motivation for courage and perseverance in the face of adversity, but also plays a pivotal role in the formation and evolution of self-concept. These changes accumulate and intensify over time, gradually contributing to an individual’s psychological development.

Early family parenting styles impact the development of *DanQi* by either addressing or neglecting the children’s psychological needs. Positive parenting styles, such as protection and encouragement, may help children develop courage and autonomy in difficult situations (Carroll, 2022; Rana and Sharma, 2024). In contrast, excessive parental control or indifference may hinder a child’s self-control and self-care (Amianto et al., 2021; Wang et al., 2022; Yan et al., 2024), which hinders the development of *DanQi*. The level of attachment between children and their parents influences the children’s level of courage as well. Adolescents who are more anxiously attached to their parents tend to have lower self-esteem and less courage (Park and Gentzler, 2023).

Adolescence marks the beginning of a new phase in DanQi development. It develops as individuals mature. The *DanQi* formed during childhood begins to significantly impact an individual’s ability to cope with adversity and setbacks. This becomes especially evident as adolescents face more complex social, academic, and physical challenges. Feedback from both home and school environments shapes *DanQi* and promotes self-awareness through external factors, such as role modeling and peer interactions. These mechanisms help individuals stay composed in challenging situations and acquire the ability to persevere despite difficulties, thereby enhancing their self-confidence. This study found that the development of *DanQi* during this period plays a critical role in forming personality, as it directly impacts the self in young adulthood. It also indirectly influences self-perception through its effect on *DanQi* in young adulthood. The impact of childhood upbringing on young adulthood self can be seen as a “springboard,” fundamentally influencing personality development (Jiang et al., 2024; Rana and Sharma, 2024). Through the gradual development of *DanQi* in adjacent stages, it is continuously strengthened, ultimately promoting the formation of self-perception in young adulthood. From a developmental psychology perspective, Erikson’s theory of self-identity emphasizes adolescence as a critical stage for integrating self-concept. When individuals fail to integrate and coordinate diverse experiences into a cohesive self, a crisis of self-identity ensues, leading to emotional challenges (Huang et al., 2025).

As individuals transition into young adulthood, *DanQi* evolves from a reaction to external challenges to a deeper influence on self-perception and personality. In the context of various choices and challenges, including academics, careers, and relationships, the courage and determination in *DanQi* form the foundation for a more resilient and independent self-concept. In this stage, individuals face external challenges while achieving psychological growth from dependence to independence and simplicity to complexity, through self-understanding, acceptance, and adjustment (Sm and Latipah, 2024). *DanQi* becomes an intrinsic motivator for self-regulation and self-affirmation, helping maintain psychological resilience and effective coping mechanisms in the face of life challenges. Individuals with greater adaptability and self-directed goal adjustment report higher well-being (Leipold et al., 2023).

The development of *DanQi* is gradual, strengthening incrementally. Each stage builds upon the previous one, and this progressive change propels the individual from dependence to independence. Throughout this process, the role of *DanQi* becomes increasingly significant. Its strength grows at each stage. This cumulative reinforcement allows early parenting styles to influence psychological traits over both short and long periods. The psychological, emotional, and cognitive changes at each stage foster the continuous development of boldness, leading to comprehensive self-growth throughout life. The influence of family and social environments on boldness is particularly strong during early upbringing, shaping self-formation through the transmission and accumulation of psychological resources. This effect is evident in later development, highlighting the significance of early parenting styles in shaping psychological traits.

Therefore, during individual growth, parents, teachers, and educators should emphasize cultivating courage, decision-making skills, psychological resilience, and self-efficacy in younger generations. These qualities will help them develop a well-rounded sense of self and acquire effective coping mechanisms for future challenges. Future research could explore programs that help parents and educators understand key factors in resilience and self-concept development in children.

## 5 Conclusion

Previous studies have demonstrated that parenting styles can impact self-development. During adolescence, parenting styles shape DanQi, which in turn influences emotional stability and autonomous decision-making during young adulthood. Thus, parenting styles that shape *DanQi* are important for developing a stable self-concept in young adulthood. We propose that *DanQi* buffers against disruption in psychological resources during adolescence and that parenting styles influence well-being through their effect on *DanQi* during the transition from adolescence to young adulthood. The results suggest that family education fostering *DanQi*, particularly in promoting courage and autonomy, is key to preventing disruptions in psychological resources during adolescence.

## 6 Limitations

This study used a self-reported segmented personality development scale, focusing on memory traces. It is crucial to recognize that using this data may introduce response biases, such as social desirability bias and recall bias, related to childhood experiences. This could impact the validity of the reported parenting styles and self-perceptions. However, while memory traces may not always reflect reality—reinforcing negative memories while overlooking positive ones—the study’s findings remain significant. This biased recollection, marked by one-sidedness and ambiguity, shapes the formation of an individual’s self-perception. This perception shapes the individual’s subjective experience and plays a crucial role in personality development. In simpler terms, the individual’s perception and emotional response to experience outweigh objective reality. Thus, despite recollection bias, these subjective perceptions and emotions remain central to shaping an individual’s personality and self-concept.

Despite the large sample size and extensive geographic coverage, this study mainly uses data from urban areas in Mainland China. This limitation may reduce the generalizability, especially for rural populations and those from different cultural contexts. Future studies should expand the sample size and include participants from more geographical locations. Another limitation is that the model only considers the link between parenting styles, *DanQi,* and self-concept, neglecting factors like genetics, social environment, and peer relationships. Self-concept is influenced by many factors and is complex. Future studies should include additional variables to clarify the role of boldness and self-concept in development.

Although this study has some limitations, it is intended to lay a foundation for future studies on the relationship between parenting styles, *DanQi,* and self-concept. Future research should improve the rigor and scope of this study to deepen understanding of the relationship between parenting styles, *DanQi,* and self-concept. These results will provide a stronger foundation for the theory and practice in the relevant fields.

## Data Availability

The raw data supporting the conclusions of this article will be made available by the authors, without undue reservation.
